# Smoking prevalence and emerging tobacco product use among Saudi adolescents: a systematic review

**DOI:** 10.3389/fpubh.2026.1850905

**Published:** 2026-07-03

**Authors:** Abdullah Alalawi

**Affiliations:** Department of Public Health, College of Health Sciences, Al-Qunfudah, Umm Al-Qura University, Makkah, Saudi Arabia

**Keywords:** adolescents, electronic cigarettes, smoking, tobacco use, waterpipe

## Abstract

**Objectives:**

To synthesize updated prevalence findings on tobacco and emerging nicotine product use, such as e-cigarettes, among Saudi adolescents based on evidence published since 2018 and to summarize reported sociodemographic and behavioral factors associated with use.

**Methods:**

A systematic review was conducted following the PRISMA 2020 guidelines. We searched PubMed, Embase, Scopus, and Web of Science for observational studies published between March 2018 and February 2026. This timeframe was chosen to update the review, which included studies published up to March 2018, and to incorporate evidence generated during the healthcare reforms introduced under Saudi Vision 2030. Study selection and data extraction were conducted using predefined eligibility criteria and standardized extraction forms. Eligible studies included Saudi adolescents aged 10–19 years reporting prevalence rates of cigarette smoking, waterpipe use, electronic cigarettes, or other nicotine products. The risk of bias was assessed using the Joanna Briggs Institute (JBI) critical appraisal checklist for cross-sectional studies. A narrative synthesis was performed.

**Results:**

Twelve studies reporting the prevalence of smoking among adolescents in Saudi Arabia were included. The prevalence of current cigarette smoking ranged from 2.8% in nationally representative surveys to 40.8% in male-only regional samples. Most mixed-gender, school-based studies reported current smoking prevalence between 8 and 23%, while ever-smoking prevalence exceeded 40% in some male-only studies. Emerging tobacco products were widely used, with e-cigarette prevalence ranging from 3 to 20.6%, and shisha use reported as high as 29%. Patterns of dual and poly-tobacco use, particularly involving e-cigarettes and hookah, were common. Across studies, males consistently demonstrated higher smoking prevalence than females. Peer smoking and family smoking were the factors most consistently associated with tobacco use across studies.

**Conclusion:**

Tobacco use among Saudi adolescents remains prevalent and varies across regions, with reported use of electronic cigarettes and dual-use patterns. Tailored prevention strategies are needed to address smoking behaviors in Saudi adolescents. National studies should be conducted to better estimate tobacco products use and other addictive behaviors among youth, guiding future prevention efforts.

**Systematic review registration:**

https://www.crd.york.ac.uk/prospero/display_record.php?ID=CRD420261320020, identifier (CRD420261320020).

## 1 Introduction

Tobacco use is one of the leading preventable causes of morbidity and mortality worldwide, resulting in over 8 million deaths annually (1). Early initiation of tobacco use during adolescence increases the likelihood of long-term nicotine dependence and is linked to a higher risk of cardiovascular disease, chronic respiratory conditions, and various malignancies later in life (2). Adolescence represents a critical developmental period during which health behaviors are established, making tobacco prevention in this age group a public health priority (3). Most adult smokers initiate tobacco use before the age of 18 years, and earlier initiation is associated with greater nicotine dependence, lower cessation success, and prolonged exposure to tobacco-related harms across the lifespan.

Globally, although conventional cigarette smoking has declined in several countries, the use of electronic nicotine delivery systems (ENDS), such as electronic cigarettes and vaping devices, has increased substantially among adolescents in many regions worldwide (4). In this review, emerging nicotine products refer to newer or increasingly marketed non-conventional nicotine delivery products, including electronic cigarettes, vaping devices, heated tobacco products, nicotine pouches, and other novel nicotine delivery systems. These products have diversified nicotine consumption patterns, raising concerns about dual use and the normalization of nicotine-related behaviors among youth (5).

In Saudi Arabia, tobacco use remains a significant public health concern despite strengthened tobacco control policies, including taxation measures and regulatory frameworks aligned with national health transformation initiatives (6). National and regional studies have reported varying prevalence estimates of cigarette and waterpipe smoking among adolescents (7, 8). A systematic review published in 2019 in the *Saudi Medical Journal* synthesized evidence on smoking prevalence among Saudi adolescents up to 2018, highlighting substantial variability across regions and study designs (9). More recently, nationally representative data from the 2007, 2010, and 2022 waves of the Global Youth Tobacco Survey demonstrated an overall decline in the prevalence of current tobacco use among adolescents aged 13–15 years, while revealing concerning trends such as earlier smoking initiation and gender-specific shifts in attitudes and quit intentions (7). Since 2018, additional epidemiological studies have been conducted, and the landscape of tobacco use among Saudi youth has evolved with an increasing uptake of electronic cigarettes and other emerging nicotine products. However, no updated systematic review has comprehensively synthesized post-2018 evidence while incorporating these emerging products.

Therefore, this review addresses the following questions: (1) What is the prevalence of tobacco use and emerging nicotine product use among Saudi adolescents in studies published since 2018? (2) What factors are associated with use, and how do patterns vary by gender, region, product type, and survey year? By narratively synthesizing prevalence estimates and associated determinants, this review seeks to inform policymakers, educators, and public health stakeholders and strengthen adolescent tobacco prevention strategies in Saudi Arabia.

## 2 Methods

This review was conducted in accordance with the Preferred Reporting Items for Systematic Reviews and Meta-Analyses (PRISMA 2020) guidelines and the Meta-analysis of Observational Studies in Epidemiology (MOOSE) recommendations (10, 11). The protocol was prospectively registered in the PROSPERO database (CRD420261320020; registered on 16 February 2026).

### 2.1 Search strategy

A comprehensive search of PubMed, Embase, Scopus, and Web of Science was conducted to identify relevant studies. The search strategy combined controlled vocabulary terms (e.g., MeSH and Emtree) with free-text keywords related to Saudi Arabia, adolescents, tobacco use, cigarette smoking, waterpipe (shisha), electronic cigarettes, and other nicotine delivery systems. Search strategies were tailored for each database. Additionally, the reference lists of the included studies were manually screened to identify additional eligible studies.

The full electronic search strategies for all databases are provided in Appendix 1.

### 2.2 Eligibility criteria

Observational studies conducted in Saudi Arabia involving adolescents aged 10–19 years were eligible if they reported the prevalence of tobacco or nicotine product use and/or examined associated determinants. Tobacco products included cigarettes, waterpipes, electronic cigarettes, and other emerging nicotine delivery systems. Only studies published in English or Arabic between March 2018 and February 2026 were included.

For studies that included participants slightly older than 19 years, inclusion was considered when the study population was predominantly adolescent, school-based, or directly relevant to the review question. Where age-disaggregated data for participants aged 10–19 years were not separately extractable, the study was retained and interpreted cautiously.

### 2.3 Definitions of key terms

In this review, “emerging nicotine products” referred to newer or increasingly marketed non-conventional nicotine delivery products, including electronic cigarettes, vaping devices, heated tobacco products, nicotine pouches, and other novel nicotine delivery systems. “Current use” was defined according to the definition used in each included study, most commonly use within the past 30 days or self-reported current use. “Ever smoking” referred to lifetime use or having ever tried smoking, even one or two puffs, as defined by the original studies.

### 2.4 Study selection

All records identified through database searches were exported to EndNote X9 for reference management, and duplicates were systematically eliminated. The author conducted title and abstract screening of all records. To reduce selection bias, an independent reviewer (NA) verified a random sample of 140 of 701 records (20.0%) at the title and abstract screening stage and all 53 full-text articles (100%) at the full-text eligibility stage. Any disagreements or uncertainties concerning study inclusion were resolved through discussion with MA.

### 2.5 Data extraction

Data were extracted using a standardized data extraction form that captured study design, setting, participant characteristics (including age and sample size), tobacco products assessed, prevalence estimates (current and ever use), and associated determinants.

Data extraction was conducted by the author using a standardized extraction form. An independent reviewer (NA) verified extracted data for all 12 included studies to ensure accuracy and minimize extraction errors. Any discrepancies were resolved through discussion.

### 2.6 Quality assessment

Methodological quality was evaluated using the revised Joanna Briggs Institute (JBI) critical appraisal tool for analytical cross-sectional studies (12). This tool evaluates key domains of risk of bias, including the clarity of inclusion criteria, the validity and reliability of exposure and outcome measurements, the identification and management of confounding factors, and the appropriateness of statistical analyses.

For overall quality classification, studies were categorized as high quality when ≥75% of applicable JBI items were fulfilled, moderate quality when 50–74% of applicable items were fulfilled, and low quality when <50% of applicable items were fulfilled. Particular attention was given to key domains related to measurement validity, confounding control, and statistical analysis.

Methodological quality was assessed by the author. To enhance reliability, an independent reviewer (NA) randomly assessed 6 of the 12 included studies (50%). Any disagreements or uncertainties were resolved through discussion with MA until consensus was reached.

### 2.7 Synthesis of results

Due to heterogeneity in study design, age ranges, outcome definitions, and tobacco product categories, a meta-analysis was not performed. Instead, the findings were synthesized narratively.

Prevalence estimates were summarized descriptively by tobacco product type and, where available, by sex. Associated determinants were extracted from each included study where reported and categorized thematically into sociodemographic, familial, peer-related, school-related, socioeconomic, and perception-based factors. Where available, adjusted estimates from multivariable analyses were prioritized over unadjusted or bivariate findings. Associations reported only in unadjusted or bivariate analyses were interpreted cautiously and were not considered equivalent to independently associated predictors.

To avoid duplication, when multiple studies analyzed the same national dataset of Global Youth Tobacco Survey (GYTS 2022), only one analysis was included.

## 3 Results

### 3.1 Search results

**Figure 1:**
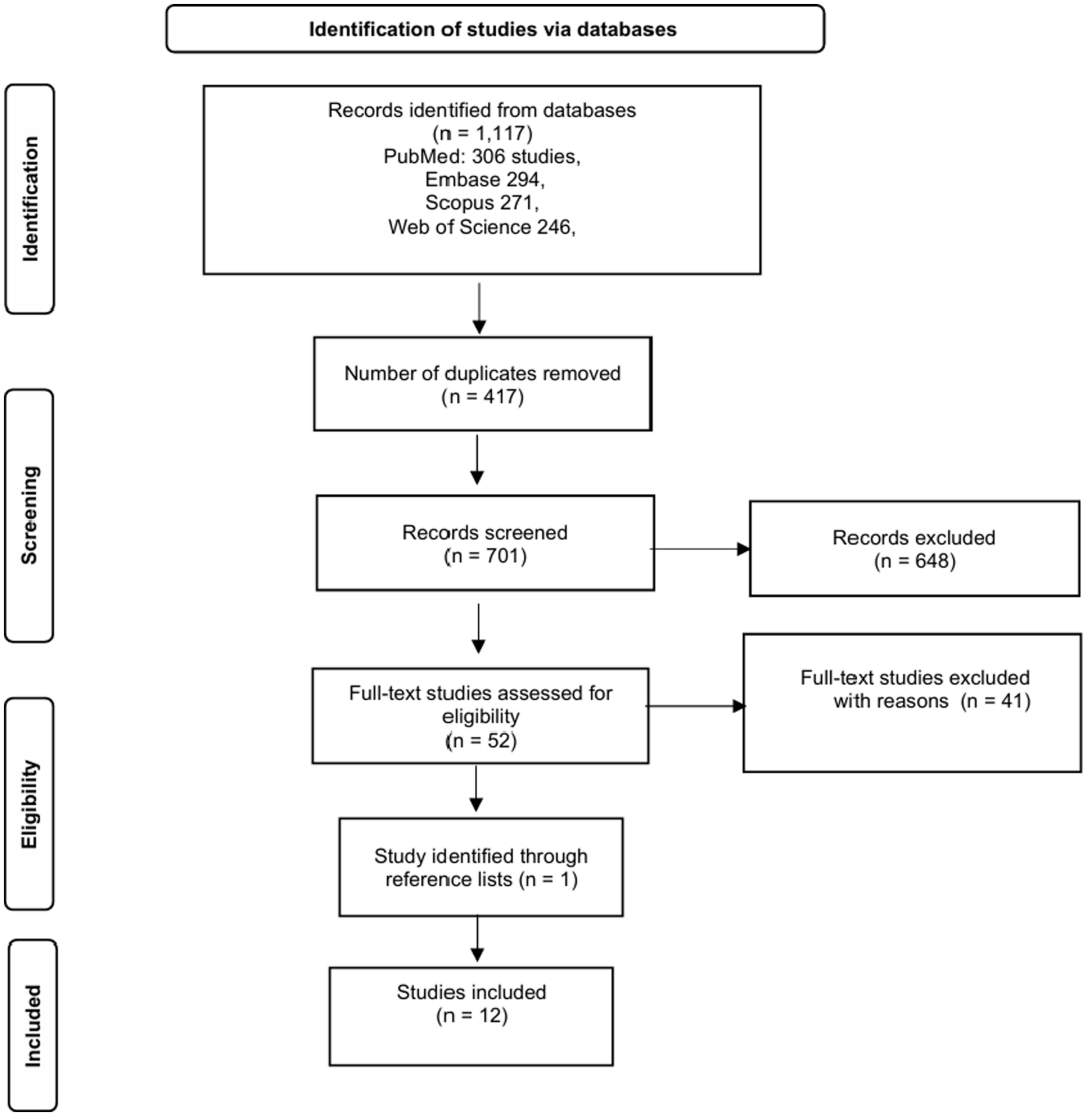
The PRISMA flow diagram was utilized to outline the study selection process in this systematic review.

The search was conducted in February 2026. The database search identified 1,117 records (PubMed: 306; Embase: 294; Scopus: 271; Web of Science: 246). One additional record was identified through reference list screening, resulting in a total of 1,118 records. After removing 417 duplicate records, 701 articles remained for title and abstract screening. Of these, 648 records were excluded for not meeting the inclusion criteria. Fifty-three full-text articles were assessed for eligibility, and 41 were excluded for reasons including non-adolescent populations, absence of relevant prevalence data, or inappropriate study design. Details of the 41 full-text exclusions and their reasons are reported in Appendix 2. Ultimately, 12 studies met the inclusion criteria and were included in the final narrative synthesis. The study selection process is presented in Figure 1.

### 3.2 Study characteristics

The included studies were conducted across multiple regions of Saudi Arabia, including Jazan (13), Arar (14), Abha and Khamis Mushait (15), Qassim (16), nationally representative GYTS data (17), Dammam (18), Jeddah (19, 20), Riyadh (21), Mecca (22), Al-Ahsa (23), and Madinah (24). Sample sizes ranged from 240 (14) to 6,717 participants (19). Five studies were school-based and included adolescents aged 12–19 years (13, 14, 19, 22, 24). Four studies were conducted exclusively among male samples (14, 18, 20, 21), while the remainder included both sexes (Table 1).

### 3.3 Prevalence of cigarette smoking

The prevalence of current cigarette smoking varied substantially across studies, ranging from 2.8% in the nationally representative GYTS survey (17) to 40.8% in a male-only regional study conducted in Arar (14).

Mixed-gender, school-based studies have reported current smoking prevalence rates ranging from 8 to 23% (13, 15, 16, 19, 24). In Jazan, the prevalence of current cigarette smoking was 14.6% (1), while in southern Saudi Arabia, it was 19.4% (15). A study conducted in Qassim reported an overall current smoking rate of 22.7%, with marked gender differences (16). In Jeddah, active smoking prevalence was 3.8% in a large sample (19), whereas another male-only study in Jeddah reported a current smoking rate of 19.1% (20).

Ever-smoking prevalence was consistently higher than current smoking prevalence. For example, ever smoking reached 45.4% among male intermediate students in Jeddah (20), and 14.1% in a large Jeddah sample including both sexes (19).

Higher prevalence estimates were consistently observed in male-only samples and vocational school settings (14, 18, 20, 21).

### 3.4 Prevalence of emerging tobacco products

Emerging tobacco products were frequently reported across the included studies (17, 21–24). The prevalence of e-cigarette use varied substantially by region. Nationally representative data indicated a prevalence of 3.0% (17), whereas a regional high school study in Mecca reported a higher estimate of 20.6% (22). In Al-Ahsa, 17.6% of male adolescents reported current e-cigarette use, including 4.8% who were dual users of conventional cigarettes (23).

In Madinah, the most common smoking pattern was the dual use of hookah and e-cigarettes (44.4%), exceeding exclusive cigarette use (24).

Waterpipe (shisha) smoking was also prevalent. In Jazan, shisha use (17.7%) slightly exceeded cigarette smoking (14.6%) (13). In Jeddah, 29.1% of male students reported having ever used shisha (20). In contrast, nationally representative data showed similar prevalence rates for current cigarette and shisha use, both at 2.8% (17).

Dual and poly-tobacco use patterns were consistently observed, particularly combinations involving e-cigarettes and hookah (22–24).

### 3.5 Gender differences

Across studies that included both sexes, males consistently demonstrated a prevalence of higher tobacco use than females (13, 16, 17, 19, 24). In Qassim, the current smoking prevalence among boys (40%) was markedly higher than among girls (5.6%) (16). Similarly, in Madinah, the overall smoking prevalence was 10.6% among males compared with 6.6% among females (24). Nationally representative data also confirmed higher tobacco use among boys (17).

Studies conducted exclusively among males reported some of the highest prevalence estimates (14, 18, 20). This suggests that variation in gender composition across studies may partly explain the observed heterogeneity in prevalence estimates.

### 3.6 Variation across study settings and populations

Prevalence estimates varied across studies conducted in different regions and population groups in Saudi Arabia. Higher estimates were reported in male-only samples, including studies conducted in northern Saudi Arabia and urban settings (14, 20, 21). In contrast, nationally representative data and large mixed-gender studies generally reported lower estimates (17, 19, 24). Because several high-prevalence studies included only male participants, observed differences should not be attributed solely to geographic variation. Instead, they likely reflect a combination of gender composition, sampling strategy, school setting, age range, product definitions, and regional context.

### 3.7 Factors associated with smoking

Several determinants have been consistently linked to tobacco use across the studies included in this review. Male sex emerged as a strong predictor (16, 17, 24). Peer smoking was identified as one of the most consistent and influential correlates (15, 23). Additionally, household and sibling smoking were repeatedly recognized as significant risk factors (15–17).

Exposure to tobacco promotion and secondhand smoke was associated with increased tobacco use (17). Furthermore, school type and socioeconomic indicators—such as maternal education and pocket money—were identified as associated factors in some studies (16, 24).

Perceptions of reduced harm from e-cigarettes were significantly associated with vaping behavior (22).

### 3.8 Quality appraisal

The methodological quality of the included studies was evaluated using the JBI critical appraisal checklist for analytical cross-sectional studies. Overall, the quality ranged from moderate to high. Seven studies were rated as high quality, (13, 15–17, 19, 22, 24) reflecting clear sampling strategies, the use of validated measurement tools, and the application of multivariable analyses to adjust for potential confounders (Table 2).

**Table 1:** Summary of the included studies.

Author (year)	Region/city	Study design	Age (years)	Sample size (n)	Product assessed	Current use (%)	Ever use (%)	Key associated factors
Alsanosy (13)	Jazan	Cross-sectional (school-based)	13–18	639	Waterpipe (Shisha) and Cigarettes	WP: 17.7%; Cig: 14.6%	Not reported (NR)	Cigarette smoking; depression patient health questionnaire-9; attitude index; age; school stage; monthly expenditure
Albangy et al. (14)	Arar	Cross-sectional (school-based)	15–18+	240	Cigarettes and shisha	Overall current smoking: 40.8%; among smokers: cigarettes 67.3%, shisha 22.4%	Ever tried smoking: 52.5%	Family member smoking: OR 3.4, 95% CI 1.9–6.1, *p* < 0.001; friends smoking: OR 5.7, 95% CI 2.8–11.7, *p* < 0.001
AlBariqi et al. (15)	Abha	Cross-sectional	16–18	568	Cigarettes	19.4%	NR	Peer smoking; household smoking; impulsivity
Vundavalli et al. (16)	Al Ras	Cross-sectional	13–15	492	Cigarettes	22.7%	NR	Sibling smoking; pocket money; academic performance
El Dalatony et al. (17)	National	Cross-sectional (GYTS 2022)	13–15	5,610	Cig; Shisha; E-cig; Heated; Smokeless	8.8% overall Boys: 10.0% Girls: 7.5%	31.5% overall Boys: 34.7% Girls: 28.0%	Male sex; Shisha; parental smoking; promotion exposure
Al-Kalif (18)	Dammam	Cross-sectional	16–19	328	Cigarettes	Current cigarette smoking: 30.2%	Past smokers: 21.3%; total smokers:51.5%	Lower health-related quality of life among current smokers (*p* < 0.01)
Mazi (19)	Jeddah	School-based cross-sectional	Grades 4–12 (mean 14.6 yrs)	6,717	Cigarettes, hookah, e-cig, smokeless tobacco and pipe.	Active smoking: 3.8%	Ever smoking: 14.1%	Older age; male sex; private school; working mother; >100 Saudi Riyal pocket money; easy access; passive smoking exposure
Alenazi (21)	Riyadh	Cross-sectional	15–21	400	Cigarettes and mixed products	27.8%	NR	No significant demographic predictors identified
Rayes (22)	Mecca	School-based cross-sectional	15–19	534	E-cigarettes	20.5%	NR	Cigarette experimentation; shisha use (AOR 9.25); living with a smoker; male; low harm perception; second-year grade
Alabdulqader et al. (23)	Al-Ahsa	Cross-sectional	12–19	472	E-cigarettes	17.6%	NR	High school level; friends who smoke; peer influence
Alzaidy (20)	Jeddah	Cross-sectional	13–15	659	Cigarettes, Shisha	14.2% (banned) vs. 23.8% (non-banned)	39.6% vs. 50.9%	School smoking bans were associated with lower smoking rates; peer smoking (p < 0.001); parental smoking (*p* = 0.007); easier access in non-banned schools (*p* = 0.003); higher passive smoke exposure in non-banned schools (p < 0.001); stronger anti-smoking attitudes in banned schools
Hakeem et al. (24)	Madinah	School-based cross-sectional	15–19	2,514	Cigarettes, hookah, e-cigarettes (dual use common)	Any tobacco use: 8.6%	NR	Male sex; maternal intermediate education; public school; lower brushing frequency

Most studies adequately described their study populations and settings. However, several relied primarily on self-reported measures and unadjusted bivariate analyses, limiting their ability to control for confounding variables. Five studies were classified as moderate quality, primarily due to limited confounding adjustment and basic statistical approaches (14, 18, 20, 21, 23).

Despite these limitations, all included studies employed appropriate cross-sectional designs and clearly reported their primary outcomes. A summary of the quality assessment is illustrated in Table 2.

**Table 2:** Quality assessment of the included studies using the JBI checklist.

Study	Selection & setting clarity	Measurement validity	Confounding control	Statistical analysis	Overall quality
Alsanosy (13)	Adequate	Adequate	Adequate	Appropriate	High
Albangy et al. (14)	Adequate	Limited	Limited	Limited	Moderate
AlBariqi et al. (15)	Adequate	Strong	Adequate	Strong	High
Vundavalli et al. (16)	Adequate	Strong	Adequate	Appropriate	High
El Dalatony et al. (17)	Strong	Strong	Strong	Appropriate	High
Al-Kalif (18)	Limited	Adequate	Limited	Basic	Moderate
Mazi (19)	Strong	Strong	Strong	Strong	High
Alzaidy (20)	Strong	Strong	Weak	Moderate	Moderate
Alenazi (21)	Strong	Adequate	Adequate	Adequate	Moderate
Rayes (22)	Adequate	Strong	Strong	Strong	High
Alabdulqader et al. (23)	Strong	Adequate	Weak	Moderate	Moderate
Hakeem et al. (24)	Strong	Strong	Strong	Strong	High

## 4 Discussion

This updated systematic review suggests that tobacco use among Saudi adolescents remains a persistent and evolving public health challenge. Although nationally representative data indicate a relatively low current smoking prevalence (2.8–8.8%) (17), several regional and male-only samples report substantially higher estimates, reaching up to 40.8% (14). Importantly, emerging nicotine products, particularly electronic cigarettes, are now widely used, with prevalence estimates reaching 20.6% in some regional studies (22, 23). Dual and poly-tobacco use patterns are frequently observed, suggesting diversification rather than a decline in nicotine exposure among youth.

The findings of this review build directly upon the previous systematic review published in the Saudi Medical Journal, which reported smoking prevalence among Saudi adolescents ranging from 9.7 to 37% prior to 2018 (9). While our findings demonstrate comparable variability, they also indicate a shift in the tobacco landscape. Unlike earlier evidence that focused primarily on conventional cigarettes and waterpipe smoking, post-2018 studies reveal a marked emergence of ENDS as a significant component of adolescent tobacco use. This suggests not merely continuity but a transformation in product preference among Saudi youth.

The nationally representative GYTS 2022 survey reported an overall tobacco use prevalence of 8.8% and an ever-use rate exceeding 30% among adolescents (17). These estimates align with those from lower regional mixed-gender samples but contrast sharply with male-only studies, which report higher prevalence rates (14, 18, 20). Similar heterogeneity has been observed in university populations, where the pooled smoking prevalence was estimated at 24.5%, with substantial variability (25). The persistent heterogeneity across adolescent and university populations suggests that tobacco use patterns are strongly influenced by regional, demographic, and institutional factors.

The increasing use of e-cigarettes among Saudi adolescents mirrors international trends. A global meta-analysis including over four million students reported a pooled current prevalence of ENDS use at 10.2% (4). Several regional estimates from Saudi Arabia meet or exceed this global benchmark (22, 23), suggesting that Saudi adolescents are following broader international patterns of vaping adoption. Furthermore, a recent Saudi systematic review specifically examining e-cigarette epidemiology reported a wide variation in prevalence (7.2–79.8%), reflecting substantial heterogeneity and the rapid evolution of use patterns (26). The consistent reporting of dual and poly-tobacco use in recent Saudi studies (23, 24) raises concerns about reinforced nicotine dependence rather than substitution or harm reduction.

Although this review included emerging nicotine products such as e-cigarettes, evidence on nicotine pouches and other newer products was limited or absent in the included adolescent studies. Therefore, the lack of findings should be interpreted as a gap in available surveillance data rather than evidence of low use among Saudi adolescents. Future national surveillance studies should explicitly assess nicotine pouches and other emerging nicotine products to better capture evolving patterns of youth nicotine use.

Consistent with earlier national evidence (9) and global findings (4), males demonstrated higher tobacco use prevalence across nearly all included studies (16, 17, 24). In some regions, the prevalence among boys was several-fold higher than among girls (16). Cultural norms, greater social mobility among males, and peer network dynamics may partially explain this disparity. However, measurable tobacco use among female adolescents indicates potential shifts in gender norms, requiring continued surveillance.

Peer smoking was the most consistently reported determinant (14, 15, 20, 21), followed by household or parental smoking (16, 17). These findings align with social learning theory and prior evidence synthesized by Alasqah et al. (9). Additionally, exposure to secondhand smoke and tobacco promotion were significant predictors in national data (17).

Notably, the perception of reduced harm associated with e-cigarettes was significantly associated with vaping behavior (22). International evidence suggests that exposure to digital media and online e-cigarette marketing is associated with subsequent initiation among adolescents (27). Although most longitudinal evidence originates outside Saudi Arabia, these findings suggest a tentative hypothesis that media exposure and harm misperceptions may contribute to ENDS uptake among Saudi youth. Future longitudinal studies are needed to examine whether these factors operate together as a causal pathway.

A cross-sectional study from Jeddah reported lower smoking prevalence in schools with smoking bans compared with schools without bans (20). However, this association should be interpreted cautiously because unmeasured confounding factors, such as socioeconomic differences, parental involvement, and school-level characteristics, may partly explain the observed difference. Given the strengthened tobacco taxation and regulatory policies implemented nationally (6), sustained enforcement of tobacco control measures and school-based preventive interventions remain critical. A recent multinational cross-sectional study among Arab university students further highlighted that interventions should target social normalization, strengthen regulatory measures, and incorporate validated behavioral assessment tools to better understand vaping-related risks among young populations (28).

The findings of this review emphasize the need for comprehensive, adolescent-focused tobacco control strategies in Saudi Arabia. Interventions should address conventional cigarettes, hookah, and e-cigarettes simultaneously, given the frequency of dual use (23, 24). Peer-centered educational programs and parental engagement strategies are warranted, given the strong influence of social networks (14, 16). Furthermore, regulatory oversight of emerging nicotine products and youth-targeted marketing should be strengthened, particularly within digital media environments (27).

Future research in Saudi Arabia should prioritize longitudinal cohort studies to better establish temporal relationships between sociodemographic, familial, peer-related, and perception-based risk factors and tobacco initiation among adolescents. Such studies are also needed to examine transitions between e-cigarette use and conventional cigarette smoking, patterns of dual use over time, and the long-term impact of recent tobacco control regulations on youth tobacco and nicotine product use.

### 4.1 Strengths and limitations

This review provides the first comprehensive synthesis of post-2018 evidence specifically focusing on Saudi adolescents aged 10–19 years and incorporating emerging nicotine products. However, all included studies were cross-sectional, which limits causal inference. The reliance on self-reported measures may introduce reporting bias. In the Saudi sociocultural context, social desirability bias and stigma surrounding youth smoking, particularly among females, may contribute to underreporting and may partly influence the observed prevalence estimates and gender differences across studies.

In addition, heterogeneity in sampling strategies, outcome definitions, and gender composition precluded meta-analysis. A further limitation is that a small number of included studies enrolled participants slightly older than 19 years, such as age ranges of 15–21 years or 15–18 + years. Because adolescent-specific data were not always separately extractable, findings from these studies may include some young adults and should be interpreted cautiously when drawing adolescent-specific conclusions.

Additionally, study screening and data extraction were primarily conducted by a single reviewer. Although 140 of 701 records, all 53 full-text articles, and extracted data for all 12 included studies were independently verified and uncertainties were resolved through discussion, the lack of fully independent dual screening at all stages may have introduced potential selection or interpretation bias. Nonetheless, consistent patterns observed across national, regional, and international comparisons strengthen confidence in the overall findings.

## 5 Conclusion

The use of tobacco and emerging nicotine products among Saudi adolescents remains a significant and heterogeneous public health concern. Although nationally representative surveys indicate moderate prevalence, regional studies and male-dominated samples reported considerably higher rates. The rising popularity of electronic cigarettes, frequent dual use, and strong social influences highlight a shifting tobacco landscape that necessitates multi-level, culturally tailored prevention strategies, including school-based programs, family engagement, and peer-focused interventions aligned with Saudi public health priorities.

## Data Availability

The original contributions presented in the study are included in the article/Supplementary material, further inquiries can be directed to the corresponding author.
