# Supplementary material for: Smoking prevalence and emerging tobacco product use among Saudi adolescents: a systematic review
Source: Front Public Health. 2026 Jul 3;14:1850905. doi: 10.3389/fpubh.2026.1850905 (PMC13375787; doi:10.3389/fpubh.2026.1850905)
Supplement: Supplementary file 1 [file Table_1.docx]

**Appendix 1.** Full electronic search strategies

Database: PubMed (MEDLINE)

("Saudi Arabia"[Mesh] OR "Saudi Arabia" OR KSA) AND ("Adolescent"[Mesh] OR adolescen* OR youth OR teen* OR "school student*" OR "high school student*") AND ("Smoking"[Mesh] OR "Tobacco Use"[Mesh] OR smoking OR tobacco OR cigarette* OR waterpipe OR shisha OR sheesha OR hookah OR "electronic cigarette*" OR e-cigarette* OR vaping OR "vape device*" OR "nicotine product*" OR "heated tobacco" OR ENDS) AND ("Prevalence"[Mesh] OR prevalence OR epidemiology OR frequency OR "cross sectional").

For Scopus / Web of Science

(TITLE-ABS-KEY("Saudi Arabia" OR KSA)) AND (TITLE-ABS-KEY(adolescen* OR youth OR teen* OR "school student*" OR "high school student*")) AND (TITLE-ABS-KEY(smoking OR tobacco OR cigarette* OR waterpipe OR shisha OR hookah OR "electronic cigarette*" OR vaping OR "nicotine product*" OR "heated tobacco" OR ENDS)) AND (TITLE-ABS-KEY(prevalence OR epidemiology OR frequency))

**Appendix 2.** Full-text articles excluded, with reasons (n = 41)

**Studies excluded because they were duplicate dataset analysis (6)**

1. Monshi SS, Desouky EDE, Aldukhail SK, Al-Zalabani AH, Alqahtani MM, Dalatony MME, et al. Tobacco use trends among youth in Saudi Arabia: 2007–2022. ***Front Public Health***. 2025;13:1608394.
2. Alshahrani NZ, Alarifi AM, Algethami MR, Aljunaid MA, Shukri AK, et al. Sex-stratified analysis of marketing exposure and current e-cigarette use among Saudi adolescents. ***Front Public Health.*** 2025;13:1649537.
3. Alshahrani NZ. Association between exposure to tobacco control measures and current nicotine use among adolescents in Saudi Arabia: evidence from the 2022 Global Youth Tobacco Survey. ***Prev Med Rep.*** 2025;59:103280.
4. Shiva Shankar B. Global Youth Tobacco Survey (2001-2022) in Saudi Arabia: an analysis of forecasting and insights pertaining to dental public health and tobacco cessation counseling. ***Cureus.*** 2025;17(6):e86525.
5. Shubayr MA, Alhazmi AS, El Dalatony MM, El Desouky ED, Al-Zalabani AH, Monshi SS, et al. Factors associated with tobacco use among Saudi Arabian youth: application of the Theory of Planned Behavior. ***Tob Induc Dis.*** 2024;22:196678.
6. Alshahrani NZ, Alarifi AM, Qarah M, Almalki S, Alshammari WH, Alnahdi RS, et al. Prevalence and correlates of ever and current dual use of cigarettes and e-cigarettes among adolescents in Saudi Arabia. ***Intern Emerg Med.*** 2026;21(1):271-280.

**Studies excluded because they were non-adolescent populations (17)**

1. Madi M, Abuohashish HM, Attia D, AlQahtani N, Alrayes N, Pavlic V, et al. Association between periodontal disease and comorbidities in Saudi's Eastern Province. ***Biomed Res Int.*** 2021;2021:5518195.
2. Althobaiti NK, Mahfouz MEM. Prevalence of electronic cigarette use in Saudi Arabia. ***Cureus.*** 2022;14(6):e25731.
3. Bin Abdulrahman KA, Alghamdi HA, Alfaleh RS, Albishri WS, Almuslamani WB, Alshakrah AM, et al. Smoking habits among college students at a public university in Riyadh, Saudi Arabia. ***Int J Environ Res Public Health.*** 2022;19(18):11557.
4. Alhemayed RS, Al-Hanawi MK. Knowledge, attitude, and practice towards e-cigarettes among adults in the Kingdom of Saudi Arabia: a cross-sectional study. ***Front Public Health.*** 2025;13:1701571.
5. Issrani R, Alruwaili DSR, Alruwaili RHG, Tasleem R, Almuaddi A, Abdelaziz KM, et al. Patterns and associated factors of shisha usage among the undergraduate students of Jouf University, Saudi Arabia: a cross-sectional study. ***Tob Induc Dis.*** 2024;22:186185.
6. Kandasamy G, Sam G, Almanasef M, Almeleebia T, Shorog E, Alshahrani AM, et al. A study on the prevalence of smoking habits among the student community in Aseer Region, Saudi Arabia. ***Front Public Health.*** 2023;11:1257131.
7. Doumi R, Khaytan S, Alobaidan AS, Alqahtany BM, Aldosari NM, Almutairi AA, et al. Knowledge, attitude, and practice of e-cigarettes of adolescents and adults in Saudi Arabia: a cross-sectional study. ***Healthcare (Basel).*** 2023;11(22):2998.
8. AlHumaidan NI, AlZelfawi LA, AlHindawi ZA, AlDosari LM, AlTowaijri AM, AlFaisal NF. Prevalence, perception, and attitude regarding electronic cigarettes usage among young adults in Riyadh, Saudi Arabia: a cross-sectional study. ***Saudi Med J.*** 2024;45(8):857-861.
9. Alkharaan H, Alrubayyi A, Kariri M, Alasqah M, Alnufaiy B, Alzahrani HG, et al. Investigating oral nicotine pouch use among adults in Riyadh, Saudi Arabia: prevalence, awareness, susceptibility, and associated symptoms. ***Front Public Health.*** 2025;13:1607656.
10. Sabbagh HJ, Khogeer LN, Hassan MHA, Allaf HK. Parental knowledge and attitude regarding e-cigarette use in Saudi Arabia and the effect of parental smoking: a cross-sectional study. ***Risk Manag Healthc Policy.*** 2020;13:1195-1205.
11. Alduraywish SA, Aldakheel FM, Alsuhaibani OS, Jabaan ADB, Alballa RS, Alrashed AW, et al. Knowledge and attitude toward e-cigarettes among first year university students in Riyadh, Saudi Arabia. ***Healthcare (Basel).*** 2023;11(4):502.
12. Alhomoud FK, Almuhayshi A, Altarouti R, Abushaheen T, Alhomoud F, Alotaibi N, et al. Electronic cigarette use in Saudi Arabia: a cross-sectional study on emerging trends and public health concerns. ***Front Public Health.*** 2025;13:1574623.
13. Daradka H, Khabour O, Alzoubi K, Nakkash R, Eissenberg T. Tobacco and waterpipe use among university students in Saudi Arabia: impact of tobacco sales ban. ***East Mediterr Health J.*** 2019;25(2):111-118.
14. Salih S, Shaban S, Athwani Z, Alyahyawi F, Alharbi S, Ageeli F, et al. Prevalence, predictors, and characteristics of waterpipe smoking among Jazan University students in Saudi Arabia: a cross-sectional study. ***Ann Glob Health.*** 2020;86(1):87.
15. Alshayban D, Joseph R. A call for effective interventions to curb shisha tobacco smoking among university students in Eastern Province, Saudi Arabia: findings from a cross-sectional study. ***Asian Pac J Cancer Prev.*** 2019;20(10):2971-2977.
16. Joseph R, Alshayban D. Changes in attitude to waterpipe tobacco smoking among youngsters in Eastern Province, Saudi Arabia: a cross-sectional study. ***Asian Pac J Cancer Prev.*** 2021;22(5):1443-1450.
17. Bin Abdulrahman KA, Khalaf AM, Bin Abbas FB, Alanezi OT. The lifestyle of Saudi medical students. ***Int J Environ Res Public Health.*** 2021;18(15):7869.

**Absence of relevant prevalence data (Wrong outcome) (16)**

1. Al-Zahrani JM, Aldiab A, Aldossari KK, Al-Ghamdi S, Batais MA, Javad S, et al. Prevalence of prediabetes, diabetes and its predictors among females in Alkharj, Saudi Arabia: a cross-sectional study. ***Ann Glob Health.*** 2019;85(1):109.
2. Alghamdi A, Fallatah A, Okal F, Felemban T, Eldigire M, Almodaimegh H. Smoking behaviour after enforcement of a 100% tax on tobacco products in Saudi Arabia: a cross-sectional study. ***East Mediterr Health J.*** 2020;26(1):39-46.
3. Alqahtani JS, Aldhahir AM, Alqarni AA, Alghamdi SM, Oyelade T, Alzaidi FN, et al. Understanding of stroke risk among smokers in Saudi Arabia. ***Medicina (Kaunas).*** 2025;61(6):1006.
4. Ramadan M, Bajunaid R, Alansari JA, Yusef H, Alsiary RA. The trends of mortality, aetiologies and risk factors of lower respiratory infections in Saudi Arabia from 1990 to 2021: results from the Global Burden of Disease Study 2021. ***J Health Popul Nutr.*** 2025;44(1):172.
5. Harakeh S, Angelakis E, Karamitros T, Bachar D, Bahijri S, Ajabnoor G, et al. Impact of smoking cessation, coffee and bread consumption on the intestinal microbial composition among Saudis: a cross-sectional study. ***PLoS One.*** 2020;15(4):e0230895.
6. Sabbagh HJ, Alamoudi RA, Khogeer LN, Allaf HK, Sait AA, Ahmed Hassan MHA. Electronic cigarettes use and parental factors among children and adolescents, Jeddah: a cross-sectional study. ***Int J Environ Health Res.*** 2025;35(8):2159-2172.
7. Sabbagh HJ, Sharton G, Almaghrabi J, Al-Malik M, Hassan Ahmed Hassan M, Helal N. Effect of environmental tobacco smoke on children's anxiety and behavior in dental clinics, Jeddah, Saudi Arabia: a cross-sectional study. ***Int J Environ Res Public Health.*** 2021;18(1):319.
8. Al-Zalabani A, Kasim K. Prevalence and predictors of adolescents' cigarette smoking in Madinah, Saudi Arabia: a school-based cross-sectional study. ***BMC Public Health.*** 2015;15:17.
9. Mazi AA. Second-hand smoke exposure among school children during COVID-19 in Jeddah. ***J Taibah Univ Med Sci.*** 2025;20(3):335-348.
10. Alghamdi A, Alghamdi H, Alghamdi A, Alghamdi A, Alghamdi A, Alkhathami A, et al. Prevalence and risk factors of urolithiasis among the population of Al-Baha Region, Saudi Arabia. ***Georgian Med News.*** 2024;(357):34-41.
11. Alduraywish SA, Alghamdi AA, Almutairi MA, Alghthami FM, Alsultan MM, Alreshaid LA, et al. Association between e-cigarette smoking and different lifestyle patterns among young adults in Saudi Arabia. ***Allergol Immunopathol (Madr).*** 2025;53(2):67-73.
12. Alnasser AHA, Al-Tawfiq JA, Kheimi RMA, Alibrahim RMS, Albanawi NAH, Almeshal AKA, et al. Gender differences in smoking attitude among Saudi medical students. ***Asian Pac J Cancer Prev.*** 2022;23(6):2089-2093.
13. Gaffar AM, Alsanosy RM, Mahfouz MS. Sociodemographic factors associated with tobacco smoking among intermediate and secondary school students in Jazan Region of Saudi Arabia. ***Subst Abuse.*** 2013;34(4):381-388.
14. Alwhaibi A, Wajid S, Alenezi A, Salami Y, Alhaydan I, Samreen S, et al. Prevalence of smoking and beliefs and attitude toward smoking habit and smoking cessation methods among pharmacy students: a cross-sectional study in Saudi Arabia. ***Front Public Health.*** 2022;10:816101.
15. Al-Nimr YM, Farhat G, Alwadey A. Factors affecting smoking initiation and cessation among Saudi women attending smoking cessation clinics. ***Sultan Qaboos Univ Med J.*** 2020;20(1):e95-e99.
16. Ahmad MS, Alslamah T, Abalkhail A, Shaik RA, Ahmad RK, Yusuf M, et al. Prevalence, patterns and contributing factors for tobacco usage amongst Saudi population: analysis from SHIS 2013. ***Eur Rev Med Pharmacol Sci.*** 2021;25(15):4909-4918.

**Inappropriate study design (3)**

1. AlAbdullah H, AlFahid AS, AlQarni A, Nazir MA. Impact of oral health educational intervention on smoking among male adolescents. ***Contemp Clin Dent.*** 2019;10(3):502-506.
2. Gaffar AM, Alsanosy RM, Mahfouz MS. Sociodemographic factors associated with tobacco smoking among intermediate and secondary school students in Jazan Region of Saudi Arabia. ***Subst Abuse.*** 2013;34(4):381-388.
3. Abid O, Alwadey AM, Eldeirawi K. Prevalence of tobacco smoking between 2009 and 2015 among students and the general population in the Kingdom of Saudi Arabia. ***Tob Induc Dis.*** 2023;21:52.
